# Chronic Inflammation as an Immunological Abnormality and Effectiveness of Exercise

**DOI:** 10.3390/biom9060223

**Published:** 2019-06-07

**Authors:** Katsuhiko Suzuki

**Affiliations:** Faculty of Sport Sciences, Waseda University, 2-579-15 Mikajima, Tokorozawa 359-1192, Japan; katsu.suzu@waseda.jp; Tel.: +81-4-2947-6898

**Keywords:** cytokine, neutrophil, macrophage, lipopolysaccharides (LPS), free fatty acids (FFA), Toll-like receptor (TLR), reactive oxygen species (ROS), anti-inflammatory effect of exercise, aging, non-communicable disease (NCD)

## Abstract

Reduced levels of physical activity in people’s daily lives cause the development of metabolic syndromes or age-related disorders. Chronic inflammation is now understood to be an underlying pathological condition in which inflammatory cells such as neutrophils and monocyte/macrophages infiltrate into fat and other tissues and accumulate when people become obese due to overeating and/or physical inactivity. Pro-inflammatory mediators such as cytokines that are secreted in excess from inflammatory cells will not only lead to the development of arteriosclerosis when they chronically affect blood vessels but also bring tissue degeneration and/or dysfunction to various organs. Chronic inflammation is also involved in sarcopenia that brings hypofunction in the elderly, dementia, osteoporosis, or cancer and negatively affects many chronic diseases and people’s healthy life expectancy. In this paper, outlines of such studies are introduced in terms of homeostatic inflammation, which occurs chronically due to the innate immune system and its abnormalities, while focusing on the efficacy of exercise from aspects of immunology and oxidative stress. The preventative effects of functional food ingredients in combination with exercise are also introduced and described. The challenges and future directions in understanding the role of exercise in the control of chronic inflammation are discussed.

## 1. Introduction

Immunity originally refers to the defense mechanism of the living body that works to maintain the state of homeostasis. It removes microorganisms or exogenous materials that enter the body while also removing abnormal endogenous materials, waste products, or diseased cells. When immune function weakens, the body becomes more susceptible to infections or becomes more likely to develop malignant tumors (cancers) [1]. On the other hand, excessive specific immune responses may lead to allergic diseases or autoimmune diseases [1,2]. Immune function that works appropriately, neither in excess or deficiency, is required to uphold homeostasis and the prevention and cure of various diseases. As such, our health is maintained greatly thanks to the appropriate function of the immune system.

The immune system consists of adaptive immunity, which is acquired after antigen stimulation and subsequent specific responses to the antigen, and innate immunity, which is rather nonspecific [3]. An immune response is initiated by pattern recognition receptors (PRRs) which recognize structural components unique to microorganisms as pathogen-associated molecular patterns (PAMPs), and the molecules released from damaging cells as damage-associated molecular patterns (DAMPs) [1,3,4,5]. DAMPs are released forms of damaged cells, and parts of DAMPs such as degraded matrix molecules, leukocyte degranulation substances, and heat shock proteins (HSPs) are considered danger signals that induce inflammatory responses [1,3,4,5,6,7]. 

Among PRRs, Toll-like receptor 4 (TLR4) plays key roles in recognizing lipopolysaccharides (LPS) and mediates signaling to produce pro-inflammatory cytokines [1,2,3,4,5]. The excessive stimulation of PRRs leads to an overproduction of pro-inflammatory cytokines and can cause systemic inflammatory response syndrome (SIRS), multiple organ failure, and sepsis [5]. Also, because of the nonspecific properties of PRRs, TLR4 recognizes endogenous substrates such as free fatty acids (FFAs) [6], HSPs [7], and inflammatory substances [8,9], and DAMPs mediate non-sterile inflammation by activating TLR4 signaling [1,2,3,4,5]. Most of these endogenous ligands are released during tissue injury and matrix degradation, leakage from necrotic cells, increased synthesis and posttranslational modification in response to inflammation, and FFAs released from adipose tissues [1,2,3,4,5,6,7,8,9]. Thus, reducing TLR4-mediated signaling and the causative ligands plays an important role in therapeutic targets for systemic inflammation.

## 2. Changes in Common Diseases and Paradigm Shift of the Immune System 

During the times before the Second World War when hygiene was poor and people were malnourished, common diseases consisted of mainly microbial infections such as pneumonia and tuberculosis, although these still occur to this day in some countries [1,2,10]. In terms of immunity, acute inflammation is essential for the prevention of such infectious diseases. Moreover, countermeasures have been implemented to cope with these diseases, including improvements in hygiene, the popularization of public nutrition, the development of drugs against causal microbes, and improved medical standards. As a consequence, Japan, for example, has become a country with exceptional longevity [10]. 

Tackling many infectious diseases is an enormous achievement by the immune system; however, the immune system can also overreact to things such as pollen, house dust, drugs, or even foods that do not used to have any pathogenicity. In other words, allergic diseases have now increased, and for example, half of the Japanese population is affected by some kind of allergic disease. The hygiene-hypothesis is famous for explaining this situation, where people have lost opportunities to become infected by pathogens because of improved hygiene and, thus, became more susceptible to allergic reactions instead [2]. Most diseases such as pollen allergies are not life-threatening, but some instances can lead to anaphylaxis, severe cases of systemic allergic reaction caused by an acquired immunity specific to allergens. 

Nowadays, malignant neoplasms (cancer), ischemic heart disease, and cerebrovascular disease account for about half of all causes of death in developed countries [10,11]. From an immunological perspective, cancer is associated with decreased immune function due to the aging of individuals [1]. On the other hand, ischemic heart disease and cerebrovascular disease are mainly considered atherosclerotic diseases, but studies have revealed that chronic systemic low-grade inflammation (hereinafter called chronic inflammation) is involved in the mechanisms of pathogenesis of these diseases, and it is well-established that chronic inflammation and oxidative stress are two major mechanisms leading to atherosclerosis [1,11]. 

Modern society has entered an age that is characterized by high fat diets, food satiation, and exercise deficiency due to automation and convenience. This has led to increases in lifestyle-related diseases including obesity, a large social problem [12,13]. Overeating and/or sedentary lifestyles can cause an accumulation of fat tissue in the body, and the infiltration of inflammatory cells such as macrophages into such tissues [1,14]. When various inflammatory mediators such as cytokines and reactive oxygen species (ROS) become produced excessively, they are released into blood circulation and affect blood vessels chronically [11]. An inflammation of the veins themselves also results in a further development of arteriosclerosis or atherosclerosis. Tissue degeneration and the malfunction of various organs also occur in a variety of etiologies [1,11,14]. 

Pro-inflammatory cytokines are suggested to be involved in diseases such as muscle atrophy that brings hypofunction to the elderly [15,16], osteoporosis caused by the activation of osteoclasts [17], cachexia that causes muscle wasting and weight-loss in chronic diseases such as cancer [18], and neurodegenerative diseases [15]. Chronic inflammation is, therefore, likely to negatively affect aging, disease susceptibility, and people’s healthy life expectancy [1,15]. Such chronic diseases are also increasing globally, with the World Health Organization (WHO) collectively naming lifestyle-related diseases, allergies, and cancers as noncommunicable diseases (NCD) and highlighting them as key issues for future medicine, healthcare, and public health [10,12,13]. 

## 3. Chronic Inflammation and Anti-Inflammatory Effects of Exercise

Chronic inflammation, as seen in NCD, does not present features that are obvious in acute inflammation [1,19,20]. Exogenous factors, such as pathogens, often initiate acute inflammation characterized by redness, swelling, fever, and pain. In contrast, endogenous materials or materials released endogenously caused by tissue damage (endogenous ligands) bind to PRRs of the innate immune system to induce chronic inflammation [1,4,11]. Mild inflammation can persist over long periods of time, causing irreversible organ dysfunction due to tissue damage and/or fibrogenesis [4,14,15]. For instance, endotoxin LPS that are constituents of enterobacteria translocate from leaky gut to the blood circulation and cause endotoxemia or sepsis [1,5,20,21,22,23]. This is a form of systemic inflammation, and it has even recently been revealed that a leaky gut is one of the pathogenic mechanisms of Alzheimer disease [24]. Also, fat tissues accumulated with obesity release FFAs upon the degradation of triglycerides [6,9]. LPS and FFAs bind to TLR4 of monocytes/macrophages and induce the production of inflammatory mediators. This means that an infiltration of inflammatory cells into the accumulated fat results in obesity becoming a site of prolonged production of inflammatory cytokines [6,9,10].

Using a mouse model of obesity that was fed a high-fat diet in a state of physical inactivity, studies have demonstrated an infiltration of inflammatory (M1) macrophages into fat tissues, the production of inflammatory cytokines, and cell necrosis/fibrosis in these mice [14,25]. However, these pathological changes that subsequently develop after chronic inflammation were preventable by exercise training [14,25]. Furthermore, a subsequent study also showed that the applicable mechanism was identified as the suppression of neutrophil infiltration into fat tissue and the inhibition of the expression of elastase or monocyte chemoattractant protein-1 (MCP-1) [26]. 

Similar to the above mechanisms, we have also reported the adaptation phenomenon in which the recruitment and activation of neutrophils were less likely to occur alongside repeated exercise [27]. These occurred alongside an absence of any increase in markers of muscle damage and were inversely correlated with stress hormone and catecholamine secretion [28]. Indeed, neutrophil ROS productivity was affected by stress [29] and aging [30]. In recent experiments using mice, muscle damage was prevented when neutrophils were depleted with anti-neutrophil antibodies, while the animals were being subjected to exhaustive exercise [31,32]. Also, the infiltration of macrophages and the production of inflammatory cytokines were shown to be suppressed by macrophage depletion [33]. We have, therefore, been studying the effects of exercise-induced anti-inflammatory effects to suppress the recruitment and activation of neutrophils and monocytes/macrophages as the sources of inflammatory mediators and ROS and in preventing exercise-induced tissue damage [5,9,33,34,35,36,37,38,39,40,41,42,43,44,45,46,47]. 

The WHO and the American College of Sports Medicine (ACSM) recommends a minimum of 150 min of moderate to vigorous physical activity (MVPA) per week for health promotion [10]. However, for example, nearly half of Japanese older adults living in rural community (46.3%) do not meet these guidelines [10]. Moreover, an optimal approach to increasing physical activity has not been developed. Promoting social participation could be a new approach to increasing physical activity for community-dwelling older adults because social participation is accompanied with going outdoor regardless of their attitude toward physical activity. There are substantial potential participants who still do not participate in social activity but are willing to participate in the activity. Hence, increasing the number of social group members may increase physical activity and prevent dementia onset [10]. A study has shown that approximately 100 min of walking exercise per week for three months in elderly people without prior exercise habits resulted in decreased markers of neutrophil activation and oxidative stress [48]. Apart from the effects on neutrophils, various exercise-induced anti-inflammatory effects on the innate immune system have been uncovered: for example, changes in M1/M2 macrophages in fat tissue due to exercise training [25] or arteriosclerosis and changes in blood monocyte subsets [49]. These studies will potentially expand from preventive medicine to preemptive medicine to reveal the effects of exercise from a pathological mechanism point of view or to develop early prognostic markers. 

On the other hand, it has been suggested that interleukin (IL)-6 is a major contributor to the anti-inflammatory effects of exercise [50]. Contracting muscles produce IL-6 and release it into circulation depending on the energy demand, and IL-6 mobilizes energy substrates in a similar manner to stress hormones. Therefore, IL-6 was considered to support energy supply and endurance performance and was, therefore, referred to as a myokine [50]. However, the induction of IL-6 occurs following high-intensity and/or endurance exercise, and it was unclear whether IL-6 is released into circulation in response to moderate exercise [20,22,39,51,52,53,54,55,56]. Indeed, the above study did not show changes in IL-6 in circulation [48].

## 4. Mechanisms Involved in the Exercise Effect 

Exercise can increase energy consumption, while preventing or improving obesity, diabetes, dyslipidemia, and hypertension [12,19,57]. Exercise is also effective in preventing atherosclerotic diseases such as ischemic heart disease or cerebrovascular disease and cancer [10,49,58]. Exercise not only affects metabolism and the cardiovascular system but also supports the maintenance/enhancement of locomotive organs. This will be beneficial for preventing sarcopenia or bone fracture caused by skeletal muscle atrophy or osteoporosis and for preventing hypofunction over time [59]. 

In a study using a mouse model of physical inactivity [16], restricting hind-limb exercise increased MCP-1 expression in the gastrocnemius muscle after a few days. After 1 week, the myofiber cross-sectional area was reduced, infiltration of macrophages and tumor necrosis factor (TNF)-α production occurred, and muscle atrophy and inflammation were induced. In contrast, when recurrent compression was applied to muscles for 30 min a day for over one week, MCP-1 production, macrophage infiltration, and TNF-α production were suppressed in the gastrocnemius muscle and muscle atrophy was prevented. A supporting experiment was also conducted in which macrophage infiltration was inhibited pharmacologically, and inflammation and muscle atrophy were not found. In a cellular experiment targeting macrophages, inhibiting TNF-α production by mechanical stress has been proven to be the basis of the anti-inflammatory effect [16]. 

Exercise improves many states of aging, including aging-associated reductions in maximal oxygen uptake, oxidative stress, and hypofunction of various organs [15]. Nevertheless, its molecular mechanisms are not clearly understood. According to the most recent review, exercise training was suggested to improve the defects in the intracellular quality control system (Proteasome, Lon protease, Autophagy, Mitophagy, and DNA repair systems) which is considered to be an underlying cause of aging [15]. If this leads to the reduction of endogenous ligands in the body, it may allow the prevention of chronic inflammation.

By continuing to exercise at intensities that will not cause acute inflammation by tissue damage such as muscle damage [60,61,62], the intracellular quality control system will become activated and may result in the suppression of chronic inflammation. Regular exercise has also been reported to increase cellular immune function and is likely to strengthen resistance against infections and cancer [58,63,64,65]. Maintaining and enhancing physical strength by exercise is, therefore, not only important for the prevention/improvement of metabolic syndrome or aging-related diseases but also important for preserving effective immune function.

## 5. Some Challenging Countermeasures of Exercise-Induced Reductions in Chronic Inflammation

Obesity and oxidative stress are major pathogeneses of nonalcoholic steatohepatitis (NASH) [4]. TLR4 is a molecular link between nutrition, lipids, and inflammation, whilst dietary fructose intake is also associated with NASH [4,9,66]. An intake of high levels of fructose results in high triglyceride levels in plasma and their deposition in liver, intestinal bacterial overgrowth, and increased intestinal permeability, leading to elevated endotoxins and the activation of TLR4 signaling [4]. In a mouse model of NASH, exercise training prevented NASH development by suppressing chronic inflammation [66] through a reduction in the downregulation of scavenger receptors [19].

A ketogenic diet (KD) is a nutritional approach which consists of high-fat and an adequate amount of protein, whereas carbohydrate is insufficient for metabolic demands and has been used for weight control, treatments of epilepsy, cancers, diabetes, pain, and inflammation and is a nutrition approach for common individuals and trained athletes alike [67]. Ketone bodies could help mitochondria to produce more ATP; thus, an adapted utilization of ketone bodies could produce more available energy than glucose utilization, indicative of successfully reduced lipogenesis and increased lipolysis and fat oxidation [67]. Indeed, KD-fed mice showed preventive effects of obesity despite consuming more calories and displayed enhanced endurance exercise capacity without aggravated muscle injury [68]. The potential mechanism can be attributed to the keto-adaptation [69,70] as evidenced by an 8-week KD remodeling the lipid metabolism profile, including fatty acid mobilization, fatty acid oxidation (FAO), and ketone body metabolism, and thus contributing to exercise capacity. Along with an enhanced FAO capacity during exhaustive exercise, a KD may also alter IL-6 synthesis and secretion profile, thus contributing to fatty acid mobilization, ketolysis, and lipolysis; preventing muscle damage; and thus subsequently contributing to endurance exercise capacity [68]. A KD also showed potential in protecting the liver and kidney from acute exercise-induced injuries and is promising for endurance exercise [68,71]. 

However, deleterious consequences can occur from a KD, as it may cause glucose intolerance, and adverse effects of a KD on the liver should also be considered. We have reported that an eight-week KD may increase intramuscular oxidative stress [69,71]. As mentioned below, certain supplementation such as green tea extract or polyphenols may alleviate exercise-induced oxidative stress, and those antioxidants may then contribute to the promotion of muscle health and exercise capacity [34,35,37,39,45,72,73]. In any case, some combined and/or restricted applications should be considered and explored.

Besides macronutrients for energy metabolism, to counter the negative impacts of exhaustive exercise-induced tissue injuries [74,75,76,77,78], nutritional approaches such as sports supplements and micronutrients administration are often adopted. Micronutrients such as antioxidants have been reported for improving endurance capacity, and such supplements are being applied in endurance competitive events [35,38,71]. Moreover, oxidative stress is also reported to be associated with exercise-induced fatigue [72,73], where such antioxidant supplementation may be beneficial.

Although pre-exercise cooling can effectively attenuate systemic inflammatory response to exhaustive exercise [21,23,77,79], post-exercise cooling may not have significant effects [76]. Fluid intake has been reported to prevent systemic induction of IL-6 and neutrophil activation markers [40,77,78], whilst intense exercise in the menstruation phase of the menstruation cycle increases systemic inflammation [79,80,81,82]. Diurnally, evening exercise induces more marked IL-6 release than morning exercise [83]. Intestinal permeability increases following exercise, and endotoxemia occurs [5,20,21,22,23,84,85], which induces systemic inflammation [86,87,88,89,90,91,92]. Therefore, other countermeasures such as intake of some functional foods for gut barrier protection, bioavailability, distribution [93,94], and appropriate immune responsiveness should be examined in future studies. 

## 6. Conclusions

Overall disease prevention/improvement by exercise and its health promotion effects has become the slogan known as “Exercise is Medicine” as a non-pharmacological therapy in many international societies related to exercise. Nevertheless, vigorous exercise that may result in muscle damage or fatigue may actually become a stress to the body and lead to acute inflammation [86,87,88,89,90,91,92], lead to increased intestinal permeability [5,20,21,22,23,84,85], or make the body susceptible to infection [64,95,96]. Chronic inflammation may be considered as a disease resulting from civilization society, in particular from overeating and physical inactivity. The anti-inflammatory effects of exercise are becoming better understood at being effective for preventing chronic inflammation. Candidate biomarkers are used to reveal the effects of exercise from a pathological point of view or to develop early prognostic markers [48,49,50,96,97,98,99]. However, further prevention of chronic diseases, improvements in quality of life, and an extension of healthy life expectancy by implementing not only exercise but also dietary supplementation and favorable lifestyle factors are anticipated to become more actively promoted in the future [100,101].
